# Host ESCRT Proteins Are Required for Bromovirus RNA Replication Compartment Assembly and Function

**DOI:** 10.1371/journal.ppat.1004742

**Published:** 2015-03-06

**Authors:** Arturo Diaz, Jiantao Zhang, Abigail Ollwerther, Xiaofeng Wang, Paul Ahlquist

**Affiliations:** 1 Institute for Molecular Virology, University of Wisconsin-Madison, Madison, Wisconsin, United States of America; 2 Department of Plant Pathology, Physiology, and Weed Science, Virginia Tech University, Blacksburg, Virginia, United States of America; 3 Howard Hughes Medical Institute, University of Wisconsin-Madison, Madison, Wisconsin, United States of America; 4 Morgridge Institute for Research, University of Wisconsin-Madison, Madison, Wisconsin, United States of America; University of Kentucky, UNITED STATES

## Abstract

Positive-strand RNA viruses genome replication invariably is associated with vesicles or other rearranged cellular membranes. Brome mosaic virus (BMV) RNA replication occurs on perinuclear endoplasmic reticulum (ER) membranes in ~70 nm vesicular invaginations (spherules). BMV RNA replication vesicles show multiple parallels with membrane-enveloped, budding retrovirus virions, whose envelopment and release depend on the host ESCRT (endosomal sorting complexes required for transport) membrane-remodeling machinery. We now find that deleting components of the ESCRT pathway results in at least two distinct BMV phenotypes. One group of genes regulate RNA replication and the frequency of viral replication complex formation, but had no effect on spherule size, while a second group of genes regulate RNA replication in a way or ways independent of spherule formation. In particular, deleting *SNF7* inhibits BMV RNA replication > 25-fold and abolishes detectable BMV spherule formation, even though the BMV RNA replication proteins accumulate and localize normally on perinuclear ER membranes. Moreover, BMV ESCRT recruitment and spherule assembly depend on different sets of protein-protein interactions from those used by multivesicular body vesicles, HIV-1 virion budding, or tomato bushy stunt virus (TBSV) spherule formation. These and other data demonstrate that BMV requires cellular ESCRT components for proper formation and function of its vesicular RNA replication compartments. The results highlight growing but diverse interactions of ESCRT factors with many viruses and viral processes, and potential value of the ESCRT pathway as a target for broad-spectrum antiviral resistance.

## Author Summary

Positive-strand RNA {(+)RNA} viruses cause numerous human, animal, and plant diseases. (+)RNA viruses reorganize host intracellular membranes to assemble their RNA replication compartments, which are mini-organelles featuring the close association of both viral and host components. To further understand the role of host components in forming such RNA replication compartments, we used brome mosaic virus (BMV), a well characterized model virus, to study some common features of (+)RNA virus RNA replication. We show that knocking out several components of the cellular Endosomal Complex Required for Transport (ESCRT) machinery resulted in parallel defects in BMV RNA replication and replication compartment formation, whereas other ESCRT components affected RNA replication independently of replication compartment formation. Deleting a subset of ESCRT proteins altered the frequency of replication compartment formation but had no effect on the size of these compartments, whereas a second subset affected RNA replication independently of replication compartment formation. Moreover, BMV’s interaction with the ESCRT machinery appears to be distinct from that reported for other viruses and from the ESCRT requirements for forming vesicles in cellular multivesicular bodies. These findings further illuminate the remarkable abilities of positive-strand RNA viruses to integrate viral and host protein functions to remodel membranes, and suggest potentially potent new ways to control such viruses.

## Introduction

A universal feature of positive-strand RNA ((+)RNA) viruses is that they multiply their RNA on intracellular membranes, usually in association with vesiculation or other membrane rearrangements [1–3]. Cellular membranes play crucial roles in RNA replication by providing necessary host factors [4–11], serving as a scaffold to localize viral components necessary for viral replication [12], and protecting viral RNA from cellular defense mechanisms [13].

One (+)RNA virus that has been studied as a model for RNA replication is brome mosaic virus (BMV), a member of the alphavirus-like superfamily of human, animal, and plant viruses. BMV is the type member of the bromoviruses, a group of icosahedral, tripartite, (+)RNA viruses that infect plants [14]. BMV can also replicate in the yeast *Saccharomyces cerevisiae* [15]. The techniques of yeast genetics and molecular biology have greatly facilitated investigation of BMV replication and host-virus interactions [5, 16, 17]. In both yeast and plant cells, BMV RNA replication depends on the viral 1a and 2a^pol^ proteins and specific *cis*-acting RNA signals [18], generates a considerable excess of positive- to negative-strand RNA [15], and efficiently directs subgenomic mRNA synthesis [15]. Additionally, in yeast as in plants, the 1a and 2a^pol^ proteins properly localize to the ER [19, 20] and viral RNA is selectively encapsidated into progeny virions [21]. In yeast, replication factor 1a localizes to the outer perinuclear ER membranes and induces 50–75 nm vesicular invaginations or spherules that, in the presence of 2a^pol^ and a replicable RNA template, serve as compartments or mini-organelles for RNA replication [22]. Highly similar spherular invaginations of the perinuclear ER membrane are prominent features of natural plant infections by BMV and its close relatives CCMV and BBMV [23, 24], and equivalent invaginations of other membranes are associated with genome replication in plant cells by other *Bromoviridae* [25], *Tymoviridae* [26, 27], *Tombusviridae* [28] and other plant viruses, and in animal cells by human- and animal-infecting alphaviruses [29–31].

The endosomal sorting complex required for transport (ESCRT) machinery is conserved from *Archaea* to animals and is responsible for the formation of intralumenal vesicles (ILVs) in the biogenesis of multivesicular bodies (MVBs) [32–38]. MVBs are specialized organelles of the endosomal system that are required for lysosomal or vacuolar degradation of membrane proteins [32]. Five ESCRT complexes are recruited sequentially and perform distinct functions in MVB formation. ESCRT-0 clusters ubiquitinated cargo and recruits ESCRT-I, ESCRT-I and ESCRT-II help remodel the membranes and recruit ESCRT-III, which in turn helps mediate membrane fission (Table 1) [35–40]. Finally, through interactions with ESCRT-III components, the AAA ATPase Vps4p and accessory proteins Vta1p, Vps60p, and Did2p help disassemble the ESCRT complexes from the membranes [33, 34, 41]. Parts of the ESCRT machinery also function during cell abscission at the final stage of cytokinesis [35, 42] and during budding of enveloped viruses from the plasma membrane [43–46]. In each case, the ESCRT machinery functions from the cytoplasmic face of the bilayer and invaginates the membrane away from the cytoplasm, thus inducing “negative” membrane curvature.

**Table 1:** ESCRT subunits and associated proteins.

ESCRT complex	Function	Yeast protein	Human protein	ESCRT activity	Biological role
0	Clustering of Ub cargo	Hse1	STAM1/2	Binds ubiquitilylated cargo	MVB biogenesis
I	Membrane budding	Vps23	TSG101	Binds Ub, ESCRT-0, Bro1 and viral proteins	MVB biogenesis, viral budding and replication, cytokinesis
II	Membrane budding	Vps36	VPS36	Binds PI containing membranes, Ub and ESCRT-I	MVB biogenesis
III	Membrane scission	Vps20	CHMP6	Binds ESCRT-II and Doa4, acts as nucleator of Snf7 polymer	MVB biogenesis
III		Snf7	CHMP4	Main driver of membrane scission, binds Bro1	MVB biogenesis, viral budding and replication, cytokinesis
III		Vps24	CHMP3	Caps Snf7 polymer, recruits Vps2	MVB biogenesis, viral budding, cytokinesis
III		Vps2	CHMP2	Recruits Vps4, initiates ESCRT disassembly	MVB biogenesis, viral budding, cytokinesis
III related	ESCRT disassembly	Did2	CHMP1	Recruits Vps4	MVB biogenesis, cytokinesis
III related		Vps60	CHMP5	Binds Vta1	MVB biogenesis
ESCRT associated		Vps4	VPS4	AAA ATPase disassembles ESCRT-III, active function in MVB membrane scission	MVB biogenesis, viral budding and replication, cytokinesis
ESCRT associated		Vta1	VTA1(LIP5)	Binds Vps4 to promote ESCRT-III recycling	MVB biogenesis, viral budding

Abbreviations: ESCRT: endosomal sorting complex required for transport; CHMP: charged multivesicular body protein; MVB: multivesicular body; Ub: Ubiquitin; DUBs: deubiquitylating enzyme; PI: phosphoinositide.

The spherular replication vesicles induced by BMV and many other (+)RNA viruses share topological similarities with ESCRT-dependent viral budding and MVB vesicles, in that the membrane is invaginated away from the cytoplasm, with a major difference being that such spherules do not pinch off and release from the membrane [22]. The assembly and function of BMV replication complexes further share similarities to the replicative cores of retroviruses and double-stranded RNA viruses [22, 47]. Previous results showed that key components of the ESCRT / MVB pathway, *DOA4* and *BRO1*, were crucial for proper BMV RNA replication [11]. However, the involvement of *DOA4* and *BRO1* in BMV RNA replication was not dependent on the ESCRT pathway's membrane-shaping functions, but rather on their regulating expression of *OLE1*, which encodes Δ9 fatty acid desaturase, and other lipid synthesis genes required for BMV RNA replication [11].

ESCRT components are recruited by tomato bushy stunt virus, another (+)RNA virus, to peroxisome membranes, where they promote RNA replication and the assembly of the replicase complex [7, 48]. We had previously shown that the reticulons, a family of morphogenic proteins that partition into and stabilize highly curved ER tubules, regulate spherule size and play crucial roles in forming and/or maintaining the proper structure and function of the BMV RNA replication compartments [4]. To determine if other host factors are required for this process, we systematically examined if components of the various ESCRT complexes are required for proper BMV RNA replication and replication complex formation. We show that knockout of several ESCRT components resulted in parallel defects in RNA replication and spherule formation, whereas other ESCRT components affected RNA replication independently of spherule formation. Unlike the reticulons, which regulate spherule size, deleting any of the four subunits of the ESCRT-III complex only altered spherule frequency and resulted in at least a 5-fold inhibition of BMV RNA replication. In particular, deleting *SNF7* inhibited BMV RNA replication by > 25-fold and blocked detectable spherule formation. Moreover, BMV’s interaction with the ESCRT machinery is distinct from that of TBSV and HIV-1, in that BMV 1a interacts strongly with major ESCRT-III effector Snf7p without the necessity for a bridging ESCRT-I protein.

## Results

### BMV RNA replication is inhibited in specific ESCRT deletion strains

**Fig 1:**
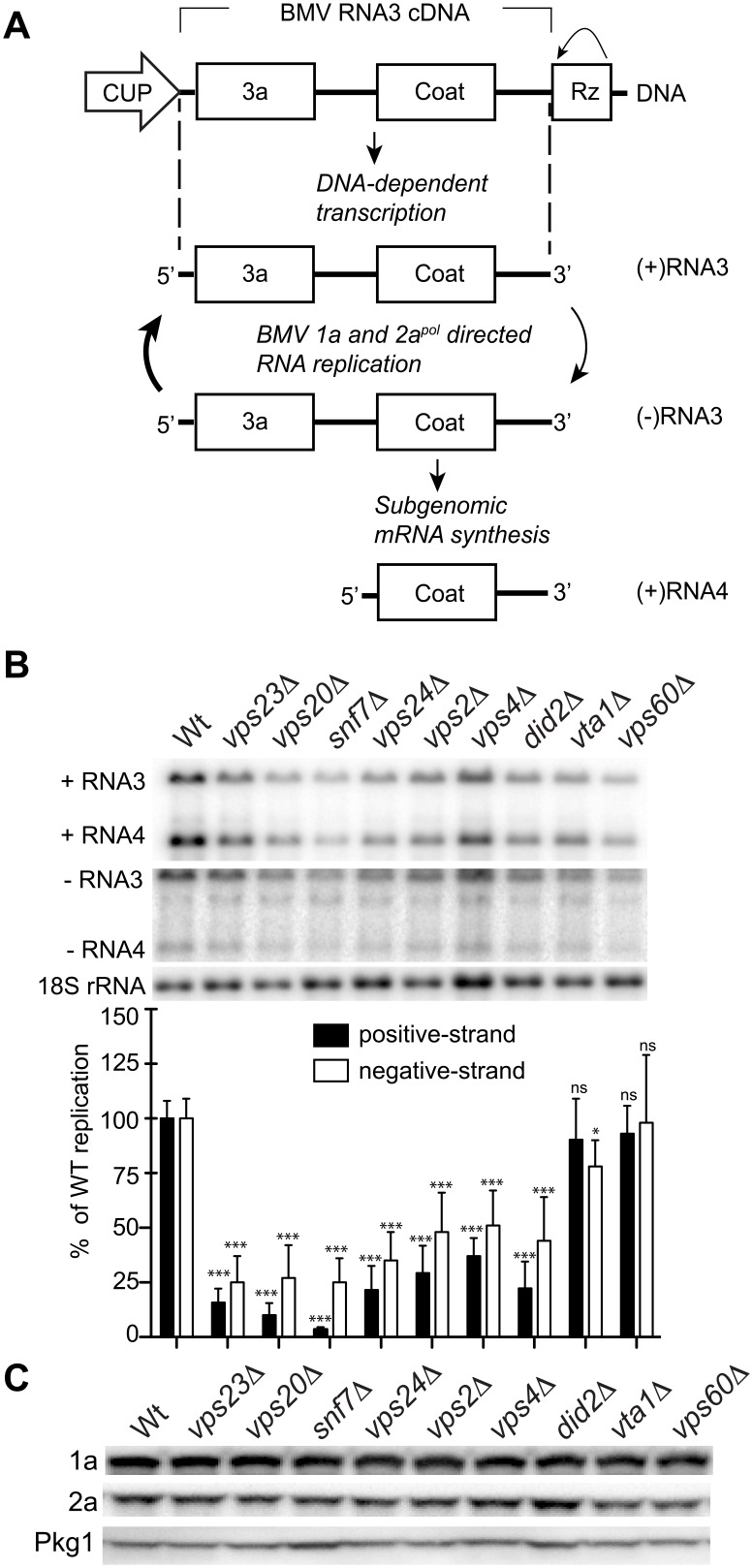
BMV RNA replication is inhibited in specific ESCRT deletion yeast strains. (A) BMV specific RNA-dependent RNA replication and subgenomic mRNA synthesis is initiated from a cDNA derivative of RNA3. DNA-dependent transcription produces an initial (+)RNA transcript that serves as a template for a 1a- and 2a^pol^-dependent RNA3 replication and sgRNA4 synthesis via a (-) RNA intermediate. (B) Total RNA extracts were obtained from wt or ESCRT deletion strains expressing 1a, 2a^pol^, and RNA3 and accumulation of negative-strand RNA3 and positive-strand subgenomic RNA4 was measured by Northern blotting. Equal loading was verified by probing for 18S ribosomal RNA. Values represent the mean of four independent experiments, with each condition tested in triplicate in each experiment. A representative blot is shown. All samples were compared to the wt control using a two tailed student t-test. * p<0.05, *** p<0.001, ns: not significant. (C) Accumulation of BMV 1a and 2a^pol^ was measured by Western blot analysis. Total proteins were extracted from equal numbers of yeast cells and analyzed by SDS/PAGE. Equal loading of total protein was verified by measuring Pgk1p levels.

Previously, Kushner et al. screened a yeast single-gene deletion library and identified **~**100 genes whose deletion inhibited or enhanced BMV RNA replication by **>**3-fold [16]. Since many of the ESCRT deletion strains either did not transform or did not grow well in the BY4743 yeast background used for the screen, we deleted individual ESCRT components in the *S*. *cerevisiae* YPH500 background. YPH500 has been used in many past studies of the assembly, structure and function of BMV RNA replication compartments [4, 22, 49–52]. Among the strains generated were deletions of components of ESCRT-0 (*hse1* Δ), ESCRT-I (*vps23* Δ), ESCRT-II (*vps36* Δ), ESCRT-III (*vps20* Δ, *snf7* Δ, *vps24* Δ, *vps2* Δ) as well as accessory proteins (*vps4*Δ, *did2* Δ, *vta1* Δ, *vps60*) (Table 1). In yeast expressing BMV 1a and 2a^pol^ proteins, plasmid-launched positive-strand RNA3 transcripts serve as templates for synthesis of negative-strand RNA3, which in turn is copied to amplify high levels of positive-strand RNA3 and to produce an additional mRNA species, subgenomic RNA4 (Fig. 1A) [15]. To determine the effects of these ESCRT gene knockouts on BMV RNA replication, we measured the accumulation of negative-strand RNA3 and positive-strand subgenomic RNA4, which are produced solely by RNA-dependent RNA synthesis, with no background from plasmid DNA-directed transcription.

Deleting components of ESCRT-0 (*hse1* Δ), ESCRT-II (*vps36* Δ), or the Vps4 accessory proteins Vta1 and Vps60 did not significantly affect either negative-strand RNA3 or positive-strand RNA4 synthesis (Figs. 1B and S1). Since they had at most minor effects on BMV RNA replication, the *vta1* Δ and *vps60* Δ strains were used as controls in experiments described in the sections below. A more pronounced effect was observed in the ESCRT-I deletion strain *vps23* Δ, resulting in six- and four-fold reductions in positive-strand RNA4 and negative-strand RNA3 levels, respectively (Fig. 1B and Table 2). In turn, deleting Vps4, the AAA ATPase that catalyzes the disassembly of the ESCRT machinery and the recycling of its subunits from membranes [53], reduced positive-strand RNA4 and negative-strand RNA3 production by three- and two-fold, respectively (Fig. 1B). Interestingly, deleting any of the four subunits of the ESCRT-III complex resulted in a 3-fold or greater inhibition of BMV RNA replication. In particular, deleting Vps20 and Snf7 inhibited positive-strand RNA4 replication by 10- and 25-fold, respectively (Fig. 1B). Thus, while multiple ESCRT components are required for efficient BMV RNA replication, the key ESCRT III effector Snf7 in particular appears to be essential for this process.

**Table 2:** Effects of ESCRT deletion on 1a-induced spherule formation and size, RNA4 replication, 1a and 2a^pol^ accumulation and localization.

Strain	ESCRT complex	Spherule Frequency (%)^a^	Avg spherule diameter (nm)^b^	(+)-strand RNA4 replication (%)^c^	(-)-strand RNA3 replication (%)^c^	1a and 2a^pol^ localization	1a accumulation (%)^c^	2a^pol^ accumulation (%)^c^
WT	-	100	67±9	100±8	100±9	ER	100±12	100±10
*vps23Δ*	I	90	68±10^ns^	16±7***	25±12***	ER	109±15 ^ns^	97±12 ^ns^
*vps20Δ*	III	77	68±8^ns^	10±6***	27±15***	ER	105±10 ^ns^	102±8 ^ns^
*snf7Δ*	III	ND	0±0***	4±1***	25±11***	ER	87±9*	93±14 ^ns^
*vps24Δ*	III	40	66±10^ns^	22±11***	35±13***	ER	100±11^ns^	110±10*
*vps2Δ*	III	80	70±12^ns^	29±12***	48±18***	ER	99±10 ^ns^	99±11 ^ns^
*vps4Δ*	disassembly	42	67±9^ns^	37±8***	51±16***	ER	93±9 ^ns^	112±16*
*vta1Δ*	disassembly	97	63±6^ns^	90±19 ^ns^	78±12*	ER	110±9 ^ns^	105±9 ^ns^
*did2Δ*	accessory	80	65±6^ns^	22x12***	44±20***	ER	80±19*	88±14*
*vps60Δ*	accessory	100	68±10^ns^	93±18 ^ns^	98±31^ns^	ER	115±13**	85±17*

^a^: (average number of spherules in ESCRT deletion cells with a clear nucleus in the plane of section among a total of two hundred and fifty cells sectioned/ average number of spherules in wt yeast cells with a clear nucleus in the plane of section among a total of two hundred and fifty cells sectioned) x 100%.

^b^: Average of independently measured diameters of 50 spherules ± standard deviation

^c^: Values represent the mean of at least three independent experiments, with each condition tested in triplicate in each experiment.

*: p < 0.05. Values compared to the wt control using a two-tailed student t-test.

**: p < 0.01

***: p < 0.001

^ns^: Not statistically significant.

To rule out that the reduced BMV RNA replication in the ESCRT mutant strains might merely be due to destabilizing effects on the viral RNA replication proteins, we measured 1a and 2a^pol^ levels in wt and ESCRT deletion yeast. Western blotting of yeast cells expressing 1a plus 2a^pol^ showed that 1a and 2a^pol^ levels were not significantly changed in the relevant strains (Fig. 1C and Table 2), indicating that the dramatic effects of multiple ESCRT factor deletions on BMV RNA replication were not due to altered levels of BMV replicase proteins.

### 1a retains ER membrane localization in the absence of ESCRTs

**Fig 2:**
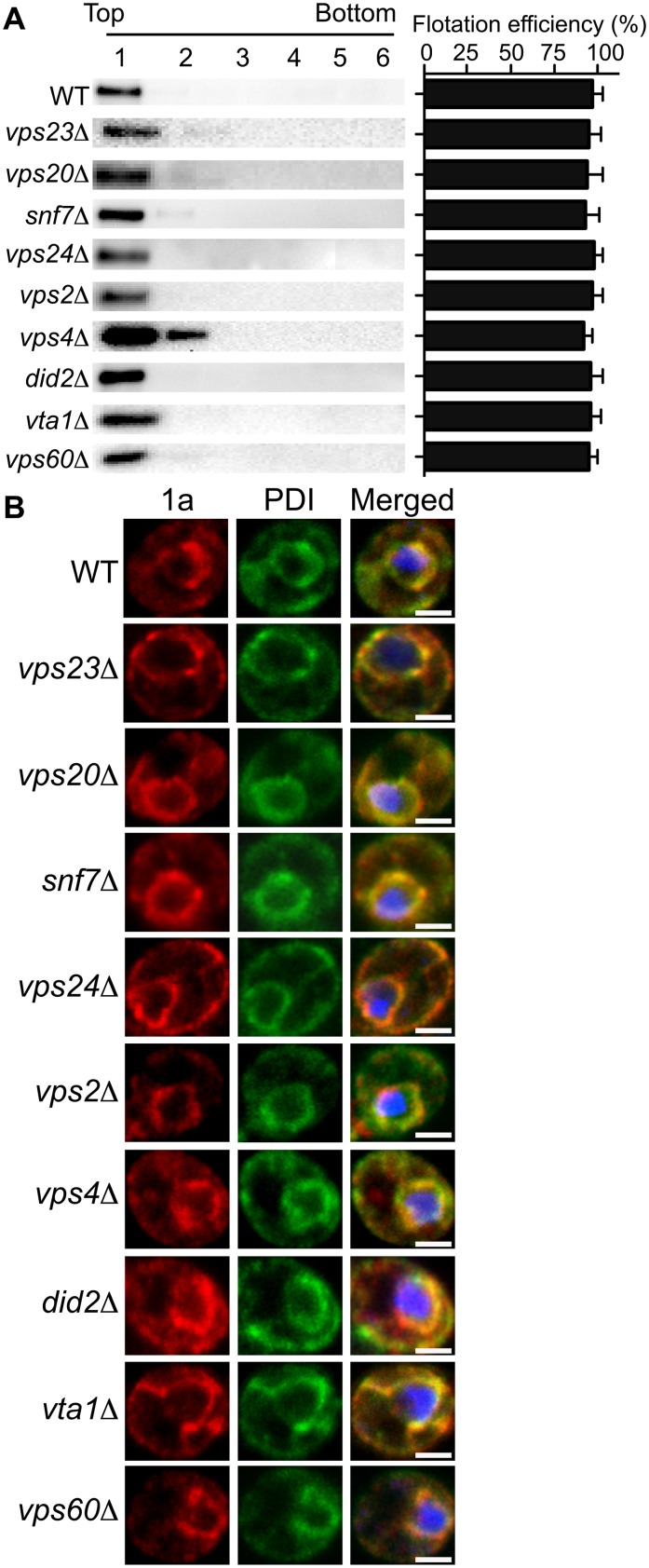
BMV 1a retains ER membrane localization in ESCRT deletion strains. (A) Membrane flotation analysis of 1a in wt or ESCRT deletion yeast strains. Representative western blots using anti-1a antiserum are shown. Membrane association was determined as the percentage of 1a present in the top two fractions. The histogram on the right shows average flotation efficiencies based on three independent experiments. (B) Confocal fluorescence images of wt and ESCRT deletion yeast cells coexpressing 1a plus 2a^pol^. Representative images for 1a (Left; red), the ER marker PDI (Center; green), and merged signals (Right) are shown. DNA was stained with DAPI (blue). Scale bars: 2 *μ*m.

To test whether the ESCRT proteins influenced 1a’s ability to associate with membranes, we performed a membrane affinity assay. Lysates of yeast cells expressing 1a in the various ESCRT deletion strains were loaded under flotation gradients, which after centrifugation were fractionated and analyzed by SDS PAGE and western blotting using an anti-1a antibody. As a measure of membrane association, flotation efficiency was determined as the percentage of total 1a protein in the gradient that was present in the top two fractions, where membranes and membrane-associated proteins fractionate. In these assays, 1a floated to the top of the gradient with the membrane fraction for all tested deletions, showing that these ESCRT factors are not required for 1a-membrane association (Fig. 2A).

In plant cells and yeast, 1a localizes predominantly at the perinuclear ER [19, 20]. To more precisely determine if 1a still localized to the perinuclear ER in the absence of the relevant ESCRT proteins, we used immunofluorescence confocal miscroscopy. In wt yeast expressing 1a and 2a^pol^, 1a was mainly associated with the perinuclear and to a lesser degree the peripheral ER membrane, and this typical localization of the 1a protein was not altered in any of the ESCRT deletion strains (Fig. 2B and Table 2).

### Snf7p interacts with 1a and relocalizes with other ESCRT proteins to BMV RNA replication sites

**Fig 3:**
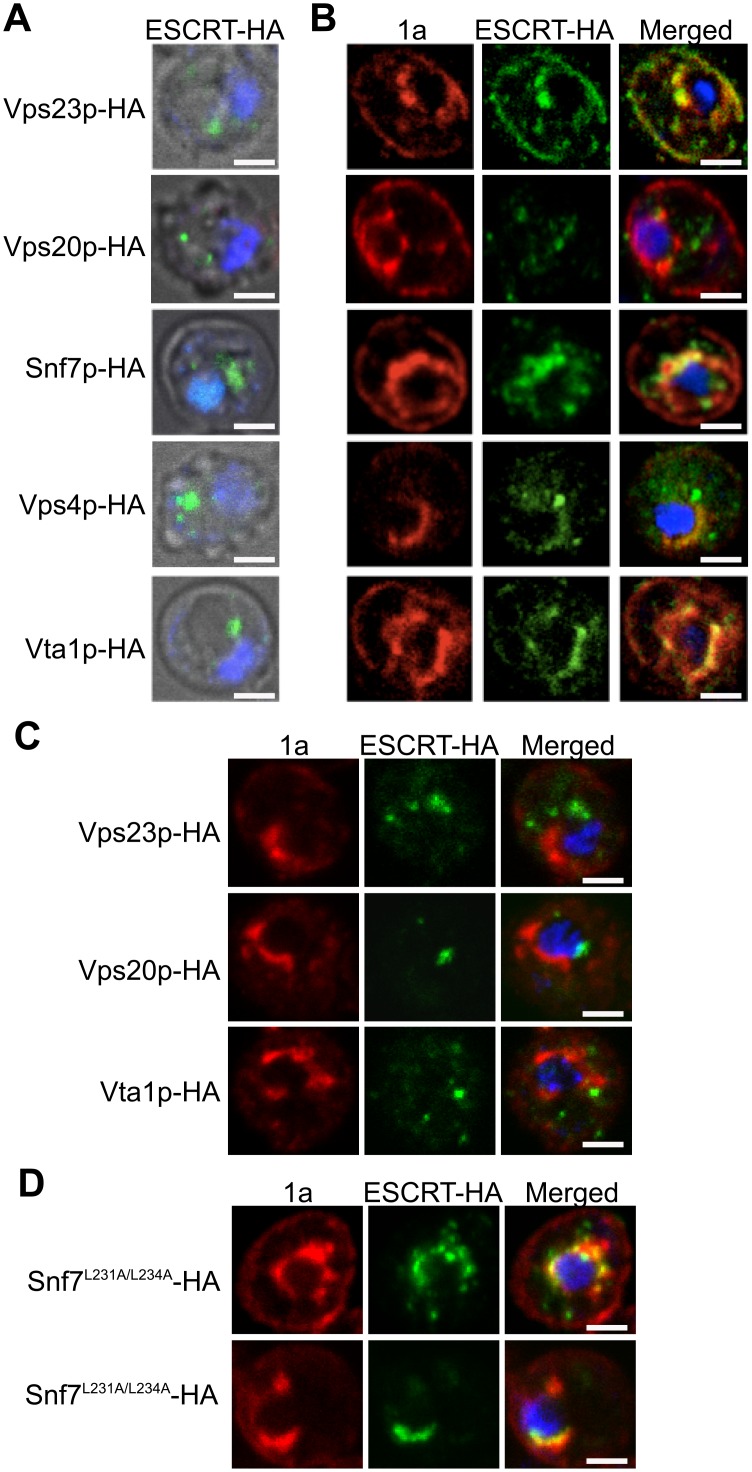
Snf7p relocalizes with other ESCRT components to the site of BMV RNA replication. Localization of HA-tagged ESCRT proteins expressed from their endogenous promoters in *vps4*Δ yeast transformed with (A) empty plasmids or (B) 1a. Yeast were immunostained for the HA-tagged ESCRT proteins (green) and BMV 1a (red) while DNA was stained with DAPI (blue). Images were cropped just outside the yeast cell wall to include only one cell. Scale bars: 2 *μ*m. (C) Localization of HA-tagged ESCRT proteins in *snf7* Δ yeast transformed with BMV 1a and Vps4^E233Q^-FLAG. Scale bars: 2 *μ*m. (D) Localization of HA-tagged Snf7^L231/L234^ proteins in *snf7* Δ yeast transformed with BMV 1a and Vps4^E233Q^-FLAG. Scale bars: 2 *μ*m.

To determine if the ESCRT proteins might be involved in forming and/or maintaining the BMV-induced membrane rearrangements, we used confocal microscopy to examine the localization of C-terminally HA-tagged versions of Vps23p, Vps20p, Snf7p, Vps4p and Vta1p expressed from their endogenous promoters from centromeric, low-copy number plasmids. Since Vps4 catalyzes ESCRT complex disassembly and recycling from membranes, we performed these confocal microscopy experiments in *vps4*Δ yeast to help stabilize any potentially transient interactions. In the absence of BMV 1a, consistent with previous reports [54, 55], ESCRT proteins were found in cytoplasmic punctate structures and were absent from the nuclear envelope (Fig. 3A). In contrast, in yeast expressing BMV 1a (Fig. 3B), the vast majority of Vps23p, Snf7p, Vps4p, and Vta1p colocalized with 1a in the perinuclear ER, the site of viral RNA replication in both plant and yeast cells [19, 20]. Interestingly, only a small percentage of Vps20p colocalized with 1a in the perinuclear ER, while the rest was found in punctate cytoplasmic structures (Fig. 3B).

**Fig 4:**
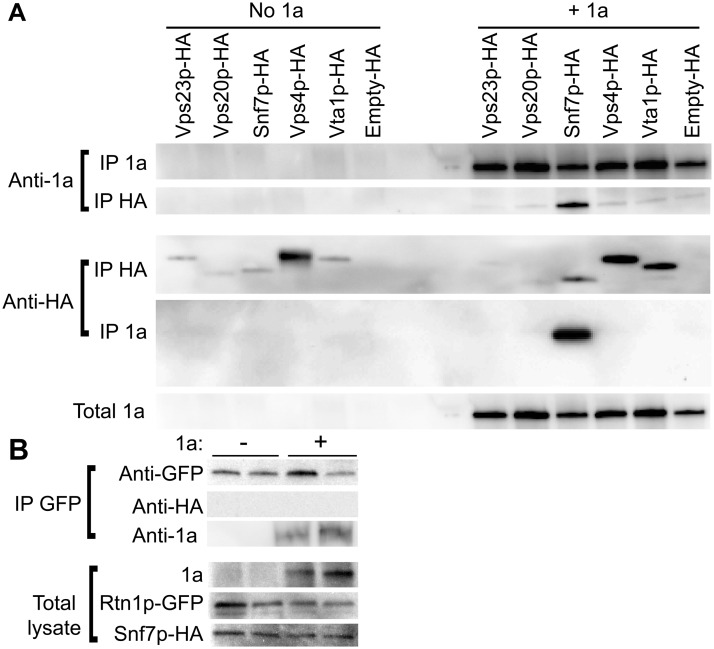
Snf7p immunoprecipitates with BMV 1a but not with Rtn1p. (A) *vps4*Δ cells expressing HA-tagged ESCRTs and either empty plasmids or 1a were lysed and the cleared lysates were subjected to immunoprecipitation using anti-1a or anti-HA antibodies (IP 1a and IP HA, respectively). The resulting immunoprecipitates were analyzed on 4–15% SDS-PAGE and immunoblotted using anti-1a or anti-HA antibodies. (B) Yeast expressing Rtn1p-GFP were transformed with plasmids encoding Snf7p-HA and either an empty plasmid or 1a. Cells were lysed, IP was performed using an anti-GFP antibody, and the resulting IPs were immunoblotted using anti-1a, anti-GFP or anti-HA antibodies. The amount of total lysate used for blotting in the lower control lanes was 1/20^th^ of the lysate volume used for the IPs.

Since expressing BMV 1a without other viral factors strikingly relocalized multiple ESCRT components (Fig. 3B), we tested whether 1a interacted with these ESCRT proteins. Accordingly, we performed coimmunoprecipitation (co-IP) assays in *vps4*Δ yeast transformed with an empty plasmid or plasmids expressing HA-tagged Vps23p, Vps20p, Snf7p, Vps4p, and Vta1p in the absence or presence of 1a. The yeast cells were lysed in buffer containing ionic (0.1% SDS) and nonionic (1% NP-40) detergents to disrupt membranes, and anti-1a or anti-HA antibodies and protein A sepharose beads were used to precipitate protein complexes. Total cell lysates and the immunoprecipitates eluted from protein A sepharose beads were subjected to SDS-PAGE and immunoblotted using anti-1a or anti-HA antibodies. As expected, on Western blots of lysates that were immunoprecipitated with anti-1a antibodies, a 1a signal was detected for all samples expressing 1a (Fig. 4A, top panel). After immunoprecipitating with anti-HA antibodies, anti-1a antibodies detected 1a only in cells co-expressing Snf7p-HA and 1a (Fig. 4A). Similarly, after immunoprecipitating with anti-1a antibodies, Snf7p-HA was the only ESCRT protein strongly detected by anti-HA antibodies (Fig. 4A). Immunoprecipitating with anti-HA antibody and immunoblotting for HA confirmed that all of the HA-tagged proteins were expressed, although Vps23-HA and Vps20-HA pulled down at lower levels in the presence of 1a (Fig. 4A, bottom panel). The results show that although several ESCRT proteins relocalize to the perinuclear ER in the presence of 1a, only the 1a interaction with Snf7 was preserved under the stringent immunoprecipitation conditions used.

Since 1a immunoprecipitates with Snf7p and we had previously shown that 1a can also interact with and relocalize the reticulons to the perinuclear ER [4], we tested if Rtn1p and Snf7p might interact. Yeast expressing GFP-tagged Rtn1 were transformed with plasmids encoding Snf7-HA and either BMV 1a or no added open reading frame. Although there was some variation in the amount of Rtn1-GFP that pulled down in the IPs, Rtn1p immunoprecipitated with 1a but no signal for Snf7p-HA was detected even in cells co-expressing BMV 1a (Fig. 4B). Since Rtn1p and Snf7p do not co-immunoprecipitate with one another, but each co-immunoprecipites with 1a, these results suggest that Snf7p and the reticulons are independently recruited by 1a to the sites of BMV RNA replication.

Although only Snf7 immunoprecipitated with BMV 1a, several other ESCRT components relocalized to the perinuclear ER in the presence of 1a (Fig. 3B). This observation suggested a possible role for Snf7p in mediating the recruitment of other ESCRT components to the perinuclear ER by 1a. To address this, we repeated the co-localization studies in *snf7*Δ yeast. As seen in Fig. 3C, Vps23p-HA, Vps20p-HA, and Vta1p-HA failed to co-localize with 1a in the perinuclear ER in the absence of Snf7p, showing that recruitment of other ESCRT components to the site of viral replication is dependent on the presence of Snf7p.

In MVB formation and virus budding, Snf7p is recruited to viral or cellular proteins through interactions with adaptor proteins such as Vps23p and Bro1p (yeast homologs of human TSG101 and ALIX, respectively) [36, 43]. Previous results show that Bro1p is required for BMV RNA replication but this involvement is not dependent on the ESCRT pathway’s membrane-shaping functions but rather on regulating expression of lipid synthesis genes required for BMV RNA replication [11]. To test for a possible role of Bro1p in bridging the Snf7p-1a interaction, two mutations were made in Snf7p (Snf7^L231A/L234A^) that are known to disrupt its interaction with Bro1p [56]. Additionally, since the interactions between 1a and ESCRT components are transient, the experiments were performed in the presence of Vps4^E233Q^, a Vps4p mutant that is incapable of mediating ESCRT-III disassembly [57]. In *snf7* Δ yeast expressing BMV 1a, Snf7^L231A/L234A^-HA and Vps4^E233Q^-FLAG, Snf7^L231A/L234A^-HA co-localized with 1a in the perinuclear ER, showing that Bro1p is not required for Snf7p to interact with 1a (Fig. 3D). This result is consistent with our previous conclusion that Bro1p’s contribution to BMV RNA replication is independent of the ESCRT pathway’s membrane-shaping functions [11].

### Deleting Snf7 abolishes spherule formation

As noted earlier, in wt yeast, expressing 1a alone induces the invagination of the outer, perinuclear ER membrane to form spherular compartments associated with BMV RNA protection and replication [22]. Intriguingly, the frequency and average diameter of these RNA replication vesicles can be changed dramatically by mutating one face of a membrane-interacting 1a amphipathic helix [50], or by knocking out the membrane-shaping host reticulon proteins [4], or a host factor promoting long chain fatty acid accumulation [52].

**Fig 5:**
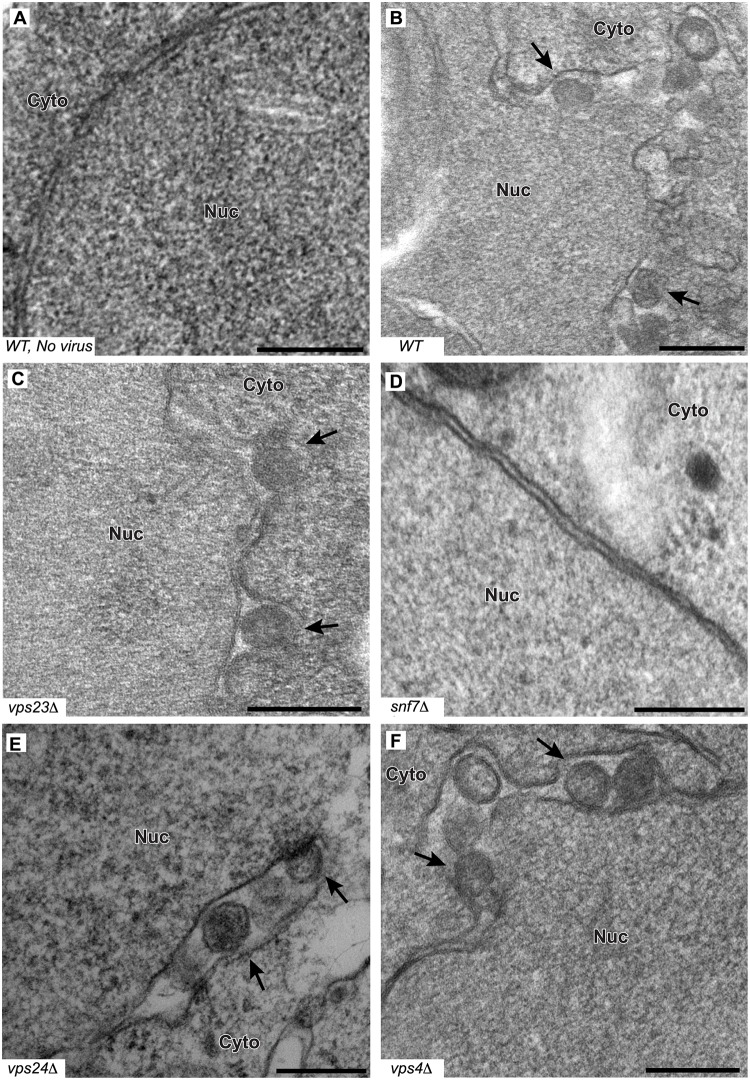
BMV spherule formation is abolished in the absence of Snf7p. Representative EM images of (A) non-infected yeast, (B) wt, (C) *vps23* Δ, (D) *snf7* Δ, (E) *vps24* Δ, and (F) *vps4*Δ yeast cells expressing BMV 1a. Black arrows point out individual spherular structures. Scale bars: 200 nm. Nuc: Nucleus, Cyto: Cytoplasm.

To determine whether the formation or structure of such spherules was affected by deleting the tested ESCRT proteins, we examined wt and single-knockout yeast by electron microscopy (EM) and measured the abundance and diameter of spherules in the subset of cells that were sectioned through the nucleus among a total of 250 cells for each strain. In wt yeast, the average diameter of spherules was 67 ± 9 nm (Fig. 5B and Table 2), similar to previous results for BMV and other *Bromoviridae* in yeast [22] and plants [23–25].

Notably, and unlike the results of depleting membrane-shaping reticulons [4], none of the tested ESCRT deletions significantly altered the distribution of spherule diameters (Fig. 5C–F and Table 2). However, deleting certain ESCRT factors reduced the number of spherules formed, which mirrors their inhibitory effects on RNA replication (Fig. 1). Most significantly, deleting SNF7 abolished detectable spherule formation (Fig. 5D and Table 2). Similarly, deleting the Snf7p oligomer capping protein Vps24p or the Snf7p recycling ATPase Vps4p, which reduced RNA4 production to 10–30% of wt, reduced spherule frequency to ~40% of that in wt cells (Fig. 5E–F). The remaining ESCRT deletions (*vps2* Δ, *20* Δ, *23* Δ and *did2* Δ), which inhibited RNA4 production to 10–33% of wt, produced spherules at 77–90% of normal frequency (Table 2). Thus, these ESCRT genes either alter the replication compartment in ways not visible at the resolution of standard electron microscopy, or are required for other step(s) of RNA replication distinct from forming the replication vesicle. Finally, *vta1* Δ, and *vps60* Δ yeast, which supported 90% of wt RNA4 production, also supported wt levels of spherule production (Table 2). Thus, while at least some ESCRT-III components and Vps4p are necessary to efficiently form spherules, they are not involved in regulating spherule size. Moreover, Snf7p was the only tested ESCRT component required for this process since detectable spherule formation was abolished in *snf7* Δ yeast.

### BMV RNA replication and spherule formation are restored by exogenous expression of ESCRT proteins

**Fig 6:**
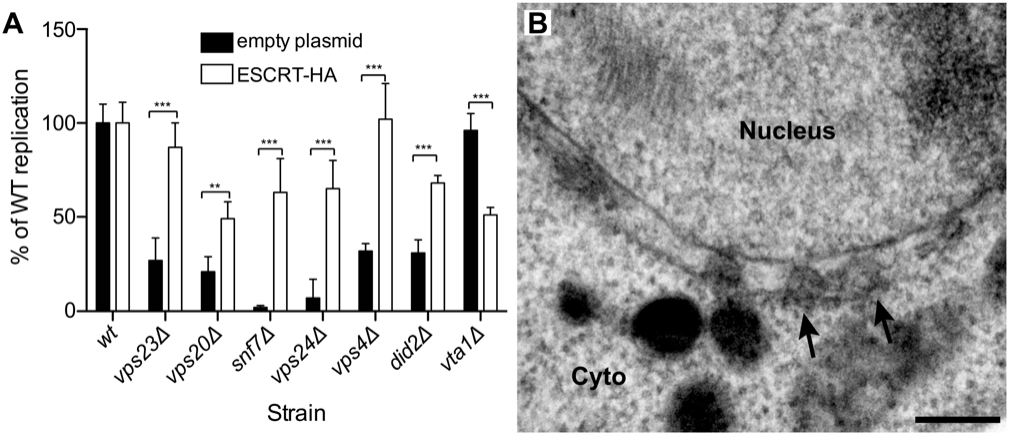
BMV RNA replication is restored in ESCRT deletion strains by supplementing HA-tagged ESCRT proteins expressed from their endogenous promoters. (A) Wt or ESCRT deletion strains were transformed with plasmids expressing 1a, 2a^pol^, RNA3 and either an empty plasmid or plasmids encoding the HA-tagged ESCRT proteins. Total RNA was extracted and accumulation of positive-strand subgenomic RNA4 was measured by Northern blotting. Values represent the mean and standard deviation of three independent experiments. *** p < 0.001. (B) Representative EM image of *snf7* Δ yeast expressing 1a, 2a^pol^, RNA3 and Snf7p-HA. Scale bar: 200 nm.

To further confirm that ESCRT components were necessary for proper BMV RNA replication, we exogenously expressed in each relevant ESCRT deletion strain a C-terminally HA-tagged version of its corresponding deleted protein from its own promoter in a low copy number plasmid, as in Fig. 3. As seen in Fig. 6A, expressing Vps4p-HA and Vps23p-HA in their respective deletion strains restored BMV RNA replication to levels similar to those obtained in wt yeast. Likewise, BMV RNA replication was substantially restored when HA-tagged Vps20p, Vps24p, and Did2p were expressed in their respective yeast deletion strains (Fig. 6A). Any residual reductions in BMV RNA replication from wt yeast might be due to impaired function of the HA-tagged protein, since in *vta1* Δ yeast, expressing Vta1p-HA reduced BMV RNA replication levels by two fold from their initially wt level. Most importantly, in *snf7* Δ yeast, expressing Snf7p-HA not only increased BMV RNA replication by ~20-fold (Fig. 6A) but also restored spherule formation in the perinuclear ER (Fig. 6B). Spherules were 62 nm in diameter and were present at ~75% frequency compared to those in wt yeast. These results support the observations above that Snf7p is crucial for both spherule formation and BMV RNA replication.

### *Arabidopsis* SNF7 restores BMV RNA replication and spherule formation in *snf7* Δ yeast

**Fig 7:**
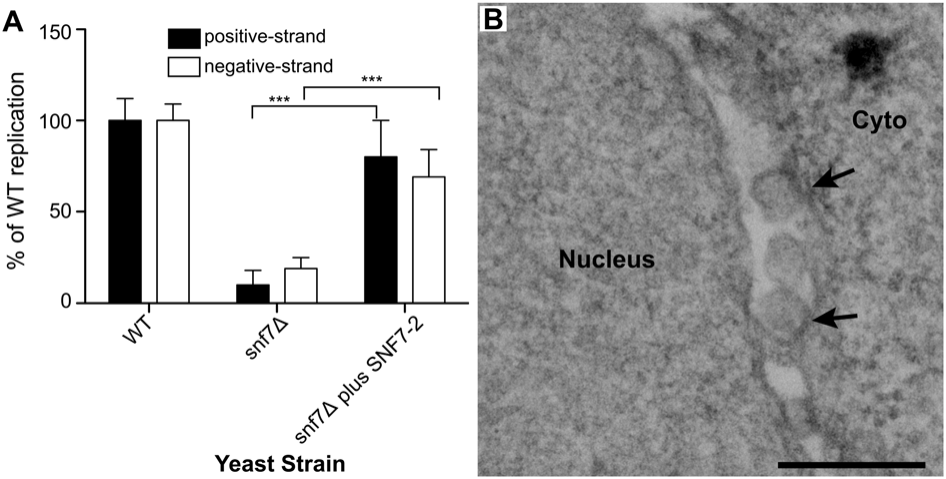
Plant homolog of Snf7p complements BMV RNA replication and spherule formation in yeast. (A) Positive-strand RNA4 and negative-strand RNA3 accumulation in *snf7* Δ yeast expressing 1a, 2a^pol^, RNA3 and either an empty plasmid or AtSNF7–2, the *Arabidopsis thaliana* homolog of yeast Snf7p, was measured by Northern blotting. Values represent the mean and standard deviation of three independent experiments. *** p < 0.001. (B) Representative EM image of *snf7* Δ yeast expressing 1a, 2a^pol^, RNA3 and AtSNF7–2. Scale bar: 200 nm.

To complement and provide a foundation for testing the effects of SNF7 on BMV RNA replication in plants, we first cloned AtSNF7–2, the *Arabidopsis thaliana* homolog of yeast Snf7p, into a yeast vector and expressed it in *snf7* Δ yeast. Wild type or *snf7* Δ yeast were transformed with plasmids expressing BMV 1a, 2a^pol^, RNA3 and either AtSNF7–2 or an empty plasmid. As shown earlier, positive-strand RNA4 and negative-strand RNA3 accumulation were severely inhibited in the absence of Snf7 (Fig. 7A). However, expressing AtSNF7–2 in *snf7* Δ yeast restored RNA4 positive-strand and negative-strand accumulation to 80% and 69% of that in wt yeast (Fig. 7A).

Since AtSNF7–2 restored BMV RNA replication, we next looked at spherule formation. While spherules were not detected in *snf7* Δ yeast among thousands of cells examined by EM (Fig. 5D), in *snf7* Δ yeast expressing AtSNF7–2, spherules with an average diameter of 66 nm were found in the perinuclear ER at ~70% of their frequency in wt yeast (Fig. 7B). Thus, *A*. *thaliana* SNF7–2 is fully compatible with BMV in restoring both BMV RNA replication and spherule formation in *snf7* Δ yeast.

### *Arabidopsis* SNF7 mutants dominantly interfere with BMV RNA replication in plants

**Fig 8:**
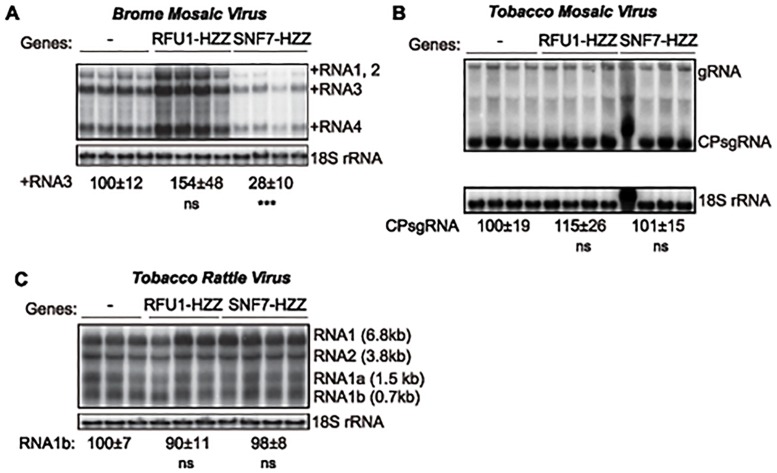
Dominant negative AtSNF7–2 inhibits BMV RNA accumulation in plants. Total RNA was extracted from *N*. *benthamiana* leaves that were co-infiltrated with Agrobacterium carrying plasmids launching an empty plasmid (-), RFU1-HZZ or AtSNF7–2 and either (A) BMV, (B) tobacco mosaic virus (TMV) or (C) tobacco rattle virus (TRV). Accumulation of genomic and subgenomic RNAs for each virus was measured by Northern blotting. Equal loading was verified by probing for 18S ribosomal RNA. Values represent the mean and standard deviation. *** p < 0.001, ns: not significant.

To test the importance of SNF7 for BMV RNA replication in plants, we turned to *N*. *benthamiana*, a systemic host for BMV infection. *Agrobacterium* infiltration of *N*. *benthamiana* plants was used to express *Arabidopsis* SNF7–2 derivatives C terminally tagged with GFP or the HZZ domain, which contains 6xHis, HA and the ZZ domain of protein A [58]. We used these tagged versions because GFP-tagged SNF7 has been shown to act as a dominant-negative inhibitor of wt SNF7 [7]. Under these conditions, BMV RNA replication was reduced by 72% in plants expressing AtSNF7-HZZ (Fig. 8A). In parallel, we expressed HZZ-tagged yeast RFU1 (regulator of free ubiquitin 1) as a control since RFU1 does not have a homolog in plants. In contrast to the inhibitory effect of AtSNF7-HZZ and AtSNF7-GFP, expressing RFU1-HZZ increased BMV RNA accumulation by ~50% compared to the empty vector.

To determine whether this inhibition by AtSNF7 mutants was specific for BMV or more generally suppresses RNA replication by other plant (+)RNA viruses, we tested tobacco mosaic virus (TMV), which replicates in the perinuclear ER [59], and tobacco rattle virus (TRV), which replicates in mitochondria [60]. Expressing RFU1-HZZ modestly increased TMV RNA replication (Fig. 8B), but overexpressing AtSN7-HZZ had no effect on either TMV or TRV RNA replication in the infiltrated leaves (Fig. 8B–C). Thus, overall, these results show that expressing AtSNF7-HZZ selectively and specifically interfered with BMV RNA replication in plants.

## Discussion

Since the assembly of BMV RNA replication complexes shares multiple similarities with budding retroviral virions [22], we investigated whether BMV spherule formation and RNA replication involved components of the ESCRT pathway. We find that deleting ESCRT pathway components results in two distinct phenotypes: one in which the RNA replication defect mirrors a defect in spherule formation, and another in which RNA replication is affected in a way or ways independent of spherule formation (Figs. 1 and 5, Table 2). This is consistent with prior observations that RNA replication requires proper spherule formation as well as other steps and factors [4, 11, 22, 49, 50]. Our results show that a subset of late ESCRT proteins, including Snf7p, Vps24p and Vps4p, are the main contributors to BMV spherule formation (Fig. 5 and Table 2). Snf7p in particular is essential for this process as it co-precipitates with BMV 1a and is recruited to the perinuclear ER (Figs. 3 and 4), it serves as a bridge to recruit other ESCRT components to the perinuclear ER (Fig. 3), and detectable spherule formation is abolished in its absence (Fig. 5 and Table 2).

Below we discuss the roles of Snf7p, Vps4p and associated factors in BMV spherule formation, how the ESCRT dependence of BMV spherule formation differs from that of MVB formation, retrovirus budding and tombusvirus spherule formation, and how some ESCRT factors contribute to BMV replication independently of spherule formation, and compare ESCRT dependence of BMV spherule formation to its dependence on host reticulons, another class of membrane-shaping proteins [4].

### Snf7-dependent, Vps23-independent BMV spherule formation

Our results show that ESCRT requirements for MVB vesicle formation and BMV spherule formation differ in important ways. MVB formation requires sequential recruitment of ESCRT-0 to cluster ubiquitinated cargo, the ESCRT-I and-II supercomplex to generate membrane invaginations into the MVB lumen, and ESCRT-III to direct membrane scission and vesicle release [36, 37]. In contrast, BMV spherule formation required ESCRT-III factors and Vps4p, but not ESCRT-0,-I or-II components (Figs. 5 and S1). This also contrasts with HIV-1 and TBSV, which require ESCRT-I factors for virion budding and spherule formation, respectively [7, 61]. HIV-1 Gag interacts with ESCRT-I factor TSG101 and ALIX (human homologs of yeast Vps23p and Bro1p) to recruit ESCRT-III factor CHMP4 (human homolog of yeast Snf7p) to HIV-1 budding sites in a process that bypasses ESCRT-II [44, 61]. Similarly, TBSV p33 interacts with Vps23p (TSG101) and Bro1p (ALIX), and these interactions appear to be required to recruit other ESCRT components to form the RNA replication compartments [7].

BMV spherule formation did not require ESCRT-0, I or II, but was sensitive to ESCRT-III components and Vps4p (Figs. 5 and S1). Most notable were the lack of BMV spherule formation (Fig. 5) and ~25-fold reduction in RNA replication (Fig. 1B) in *snf7* Δ yeast. Both spherule formation and BMV RNA replication in *snf7* Δ yeast were restored upon ectopic expression of HA-tagged Snf7p (Fig. 6), confirming the origin of these striking defects. Interestingly, in the absence of Snf7p there was a less pronounced effect on negative-strand than on positive-strand synthesis. Among many possible reasons for this, negative-strand synthesis might begin concomitantly with or soon after 1a initiates membrane invagination, but positive-strand synthesis might not proceed until the vesicle is fully enclosed. Importantly, the critical importance of Snf7p for BMV RNA replication is not restricted to yeast, as overexpressing a dominant-negative mutant of *Arabidopsis* homolog SNF7–2 in *N*. *benthamiana* plants inhibited BMV RNA replication, but had no effect on two other plant positive-strand RNA viruses (Fig. 8). In keeping with these results, wt *Arabidopsis* SNF7–2 restored BMV spherule formation and RNA replication in *snf7* Δ yeast (Fig. 7). Consistent with a direct role in spherule formation, upon BMV 1a expression, Snf7p re-localized to the perinuclear ER and was the only ESCRT component to co-immunoprecipitate with 1a (Figs. 3 and 4).

Unlike HIV-1 or TBSV, loss of TSG101 homolog Vps23p did not affect BMV spherule formation (Fig. 5C and Table 2). Moreover, while HIV-1 and TBSV recruit CHMP4/Snf7p and possibly other ESCRT-III factors through bridging interactions with the ESCRT-I factor TSG101/Vps23p, Vps23p did not pull down any detectable 1a under the same conditions that showed strong Snf7p-1a co-immunoprecipitation (Fig. 4A). Thus, while Vps23p also re-localized to the perinuclear ER in the presence of 1a (Fig. 3A, B), any Vps23p-1a interaction is indirect, transient and/or weak (Fig. 4A). Absence of demonstrable 1a-Vps23p interaction is not surprising since 1a lacks any of the L-domains previously characterized in HIV-1 and other retroviruses. Consistent with the notion that 1a does not interact with Vps23p directly and/or stably, our data further shows that Vps23p recruitment to the perinuclear ER and co-localization with 1a is dependent on Snf7p (Fig. 3C). Thus, BMV must initiate ESCRT-III recruitment and spherule vesicle assembly using different protein-protein interactions from those of MVB vesicles, HIV-1 virion budding and TBSV spherule formation. Such variations in the nature of ESCRT interactions might explain in part why BMV spherules remain connected to their parent membrane, unlike MVB vesicles and HIV-1 virions.

### Roles of other late ESCRT factors in BMV spherule formation

Yeast ESCRT-III factors Vps20p, Snf7p, Vps24p, and Vps2p are sequentially recruited to membranes and *in vitro* function together to deform membranes [40], including forming intralumenal vesicles [37]. Thus, in addition to the block to spherule formation in *snf7* Δ yeast, the 2.5-fold reduced frequency of spherules in *vps24* Δ and *vps4*Δ yeast (Fig. 5) argues for the active roles of these proteins in spherule formation, and the lesser reductions in *vps20* Δ, *vps2* Δ, and *did2* Δ yeast suggest accessory roles. In keeping with this, BMV RNA replication was reduced ~ 3- to 5-fold in these strains (Fig. 1B and Table 2). Overall, the variable importance of different ESCRT-III components for BMV spherule formation is similar to HIV-1 virion budding, for which only Snf7p (CHMP4) and Vps2p (CHMP2) are strongly required, while other ESCRT-III proteins have much lesser effects [43].

The likely roles of Vps2p, Vps20p, Vps24p and Vps4p in BMV spherule formation are suggested by their known functions and interactions with Snf7p: Vps20p triggers polymerization of coiled, membrane-deforming Snf7p filaments that are capped by Vps24p and Vps2p, which in turn recruit Vps4p for ESCRT-III disassembly and recycling [62]. Thus, Vps24p capping of Snf7p filaments may enhance their function in spherule formation. Similarly, Vps20p may promote BMV spherule formation by helping to activate Snf7p filament assembly, although reduced but continuing spherule formation in *vps20* Δ yeast implies an alternate trigger for Snf7p assembly, possibly through a 1a-Snf7p interaction (Fig. 4A).

Vps4p might contribute to BMV spherule formation in at least two ways. First, Vps4p-mediated disassembly is presumably required to recycle ESCRT-III factors to form new spherules. Residual spherule formation in *vps4*Δ yeast may occur because these dividing cells actively produce new ESCRT-III factors. Second, multiple results suggest that the Vps4p ATPase plays more active functions than simply recycling ESCRT factors, as in contributing energy for membrane deformation or scission [63, 64]. In turn, decreased spherule formation in the absence of Vps24p, Vps2p and Did2p may be due to their contributions to recruiting Vps4p (Table 1). Interestingly, the roles of Vps4p in TBSV and BMV spherule formation differ in that for TBSV *vps4*Δ yeast only formed vestigial, crescent-shaped membrane deformations [48], while for BMV *vps4*Δ yeast supported a reduced frequency of spherules of normal shape and size (Fig. 5F).

### ESCRT roles in BMV RNA replication independent of spherule formation

Although Vps23p is dispensable for BMV spherule formation (unlike TBSV), *VPS23* deletion reduced BMV RNA replication ~6-fold (Fig. 1B). Similarly, *VPS20* and *DID2* deletion induced much greater reductions in BMV RNA replication than spherule formation (Figs. 1B, 5 and Table 2). While the specific roles of these ESCRT factors in RNA replication are not yet clear, one potentially unifying explanation might involve their interactions with the Doa4p deubiquitinase. We previously showed that deleting *DOA4* or its activator *BRO1* suppresses BMV RNA replication not due to their links to ESCRT membrane-shaping functions, but rather to depleting free ubiquitin and thereby inhibiting transcriptional induction of certain lipid synthesis genes required for functional lipid composition of the membranes surrounding spherules [11, 65]. Both Vps23p and Vps20p bind Doa4p [66, 67], and mutating Vps20p’s Doa4 binding site exacerbates the deubiquitination defect in cells lacking Bro1 [67]. Thus Vps20p and perhaps Vps23p may help to activate Doa4p’s deubiquitinase activity to support BMV RNA replication. Similarly, while a detailed mechanism awaits further details, Did2p was originally identified as the site of a mutation that suppressed Doa4p mutational defects [68].

### Potential model for BMV spherule formation

Based on insights from electron microscope tomography, a new model for HIV budding was proposed in which HIV-1 Gag accumulates at the membrane, forming a partial shell that leads to plasma membrane invagination. Subsequently, Gag recruits ESCRT-I and—III factors, which constrict the initially wide membrane rim induced by Gag assembly, contributing further membrane bending to complete formation and budding of a full, vesicular membrane envelope lined by a partial Gag shell [69].

**Fig 9:**
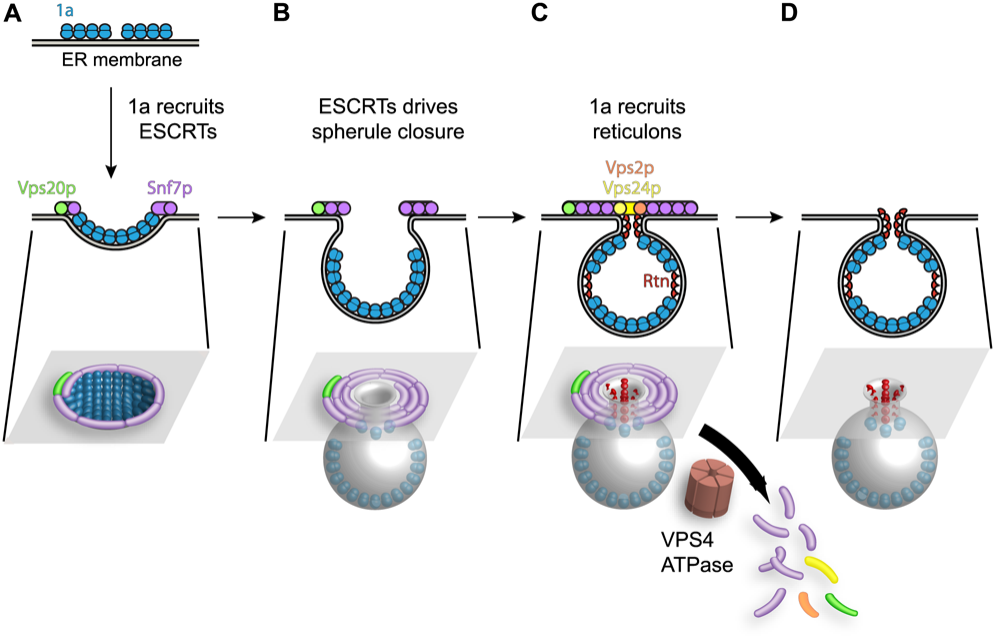
Model for the contribution of Snf7p and other ESCRT-III components to BMV spherule formation. (A) BMV 1a multimerization initiates perinuclear ER membrane invagination away from the cytoplasm, followed by recruitment of ESCRT proteins away from MVBs to the site of BMV RNA replication. (B) The concentric spirals formed by ESCRT-III components serve to constrict the wide membrane rim induced by 1a to bring the membrane lipids into close proximity to form the neck of the vesicle. (C) Unlike its natural role in MVBs where ESCRT-III continues to constrict the vesicle neck to efficiently cleave the bud to form intralumenal vesicles, either 1a, the reticulons (Rtn), which have been previously implicated in stabilizing spherule necks, or an unidentified protein prevent ESCRT-III from achieving membrane fusion, leaving an intact neck structure. (D) The ATPase Vps4p uses the energy of ATP hydrolysis to catalyze the disassembly of the ESCRT machinery and the recycling of its subunits from the membrane, leaving a spherule neck stably attached to the ER membrane.

Similarly, our recent results show that 1a parallels HIV-1 Gag in achieving high-level multimerization on the ER membrane, and implicate this 1a multimerization as one critical driver of membrane invagination [49], perhaps explaining why ESCRT-I and ESCRT-II are dispensable for BMV spherule formation (Fig. 5). A model combining these results on 1a multimerization [49] and reticulon interaction [4] with the present ESCRT results is shown in Fig. 9. Accordingly, 1a multimerization would initiate perinuclear ER membrane invagination away from the cytoplasm (Fig. 9A), followed by recruitment of Snf7p and other ESCRT-III components to constrict the wide membrane rim induced by 1a, further forming the vesicular spherule body and narrowing the vesicle neck to its final dimensions (Fig. 9B). Snf7p assembles coiled filaments that constrict membranes [70], and most mechanistic models envision that membrane constriction occurs as the filaments slide past each other to tighten and close the coil [71].

While ESCRT-III constriction continues to cleave the vesicle neck to release MVB intralumenal vesicles and HIV-1 virions, BMV avoids this step, leaving the spherule neck stably attached to the ER membrane as neck scission might be blocked by 1a, host factors or both. One possibility shown in Fig. 9C is that neck scission is blocked by host reticulons, another class of host membrane-shaping proteins that are independently recruited by 1a (Fig. 4B), stabilize positive membrane curvature as in spherule necks, and are required for BMV spherule formation and RNA replication [4]. Consistent with this model, protein scaffolds that stabilize high membrane curvature [72], like those formed by the reticulons (Rtn), inhibit membrane fission [73].

We had previously shown that the reticulons coimmunoprecipitate with 1a and are recruited to the interior of the spherules. Moreover, since the reticulons regulate spherule size and play crucial roles in forming and/or maintaining the proper structure and function of the BMV replication compartments, they remain stably associated within the spherules (Fig. 9D and [4]). In contrast, the association of ESCRT components with spherules is transient, since visualizing these associations required deleting *VPS4* (Figs. 3 and 4). Thus, in the presence of active Vps4p, ESCRT components are released from spherules and recycled as for MVBs. Interestingly, while modulating reticulon levels alters the diameter of BMV spherules [4], deleting ESCRT genes only modulated the number of spherules formed, with no significant effect on their size (Fig. 5 and Table 2). This responsiveness of spherule size correlates and is likely mechanistically linked to the stable association of the curvature-modulating reticulons with BMV spherules, compared to the transient association of ESCRT components during spherule formation. Further understanding of the detailed nature and ultrastructure of these interactions should provide additional means to control and potentially to use the remarkable abilities of positive-strand RNA viruses to remodel membranes and to selectively capture and replicate RNAs.

## Materials and Methods

### Yeast and plasmids

Yeast strain YPH500 and culture conditions were as described previously [15]. The following strains were generated for this work: *hse1*Δ (YPH500 *hse1*::*kanMX4*), *vps23*Δ (YPH500 *vps23*::*kanMX4*), *vps36*Δ (YPH500 *vps36*::*kanMX4*), *vps20*Δ (YPH500 *vps20*::*kanMX4*), *snf7*Δ (YPH500 *snf7*::*kanMX4)*, *vps24*Δ (YPH500 *vps24*::*kanMX4*), *vps2*Δ (YPH500 *vps2*::*kanMX4*), *vps4*Δ (YPH500 *vps4*::*kanMX4*), *vps60*Δ (YPH500 *vps60*::*kanMX4*), *did2*Δ (YPH500 *did2*::*kanMX4*), *vta1*Δ (YPH500 *vta1*::*kanMX4*). Genomic replacements were made using amplified *KanMX4* cassettes flanked by 5’ and 3’ homologous recombination regions. The strain expressing the chromosomal alleles of *RTN1* as a GFP fusion was obtained from Invitrogen (Carlsbad, CA). For endogenous expression of ESCRT proteins, the coding region of the full-length protein plus the ~500 base-pair upstream sequences were PCR amplified from wt yeast DNA with appropriate primers and inserted into a CEN plasmid. An HA- or a FLAG-tag was added at the C-terminus of the ESCRT ORFs. Snf7^L231A/L234A^-HA and Vps4^E233Q^-FLAG were created by site directed mutagenesis.

To express *Arabidopsis thaliana* SNF7–2 in the yeast *snf7Δ* mutant strain, AtSNF7–2 cDNA was amplified by PCR and inserted into ycpLAC33, a CEN plasmid. An HA tag sequence is attached to the 3’ end of AtSNF7–2 coding sequence and expression is under the control of the *GAL1* promoter. Plasmids expressing the dominant negative mutant of AtSNF7–2, SNF7-HZZ, was made by adding the sequence of the HZZ multitag from pBG1805 at the 3’ end of AtSNF7–2 using PCR. The multitag from pBG1805 contains 6×His, HA, and the ZZ domain of protein A [58]. AtSNF7-HZZ was cloned into agrobacterium binary vector pCAMBIA1300 under the control of an enhanced CaMV 35S promoter to make pAG2P-S7-HZZ. To make pMDC-RFU1-HZZ to overexpress yeast RFU1 in plants, the RFU1 gene in pBG1805 (a Gateway destination vector) was mobilized to a Gateway entry vector first and then to the Gateway-based binary vector pMDC32 [74], which expresses target genes using the enhanced CaMV 35S promoter. BMV 1a was expressed under control of the *GAL1* promoter using pB1YT3L, a pB3YT3 [75] derivative with a *LEU* marker, 2a^pol^ was expressed from pB2CT15 (*ADH1* promoter) [15] and BMV RNA3 was expressed under control of a *CUP1* promoter from pB3VG128H, or under control of a *GAL1* promoter from pB3MS82, both RNA3 derivatives have a four-nucleotide insertion in the coat protein gene that abolishes expression of the coat protein [76]. To make plasmids launching BMV RNAs mediated by agrobacterium infiltration, overlapping PCR was used to add a CaMV 35S promoter sequence to the 5’ end and a ribozyme from hepatitis delta virus to the 3’ end of the cDNAs of BMV. The BMV launching cassettes were then cloned into pCAMBIA1300 via *EcoRI* and *PstI* digestions. Plasmids launching tobacco mosaic virus (TMV) and tobacco rattle virus (TRV) by agroinfiltration were pTRBO [77] and pTRV-1 and-2 [78].

### BMV replication assays in plants

*N*. *benthamiana* plants were grown in a growth chamber under long day conditions (16 h light and 8 h dark) at 26°C. Agrobacterium infiltrations were performed following a protocol described by Bendahmane et al. 2000 [79]. Agrobacteria cultures were grown at 30°C in medium with kanamycin (50 mg/L), 10 mM MES (morpholine ethanesulfonic acid, ph 5.9), and 50 uM acetosynringone. These were subcultured once and harvested when the OD_600_ reached 0.8–1.0. Agrobacterial cells were brought to OD_600_ = 1.0 in the infiltration solution (10 mM MgCl2, 10 mM MES, pH 5.9, and 150uM acetosynringone) and incubated at room temperature for at least 3 hours. These cultures were then mixed for co-infiltration suspensions with final concentrations listed as followings: BMV RNA1, 2, or 3 at OD_600_ = 0.05; pTRBO, pTRV-1 and pTRV-2 at OD_600_ = 0.25; pMD32-RFU1, or pAG2P-HZZ at OD_600_ = 0.5. The aforementioned culture combinations were co-infiltrated into 8-week-old plants. After 48 hours, the infiltrated leaves were harvested for RNA extraction. Plants infiltrated with BMV only or co-infiltrated with pMDC32-RFU1-HZZ were used as controls. For the Northern blot in Fig. 8B, the first lane of the SNF7-HZZ samples was excluded from quantitative analysis since the RNAs migrated more slowly through the gel due to some salt contamination.

### Membrane flotation assay

Ten OD_600_ units of yeast cells grown to mid-logarithmic phase were spheroplasted and resuspended in 350 μl buffer TNE (50 mM Tris-HCl (pH 7.4), 150 mM NaCl, 5 mM EDTA, 5 mM benzamidine, 1 mM PMSF, and 10 μg/ml each aprotinin, leupeptin, and pepstatin A). Spheroplasts were lysed via 25 passes through a 22 gauge, 4 cm long needle. Total lysates were centrifuged for 5 minutes at 4°C at 500×g to remove cell debris, and 250 μl of supernatants were mixed with 500 μl of 60% Optiprep (Axis-Shield, Oslo, Norway). Density gradient centrifugation was performed for 2 hours at 55,000 rpm in a Beckman TLS55 rotor using 600 μl of each sample overlaid by 1.4 ml of 30% Optiprep and 100 μl of lysis buffer [80]. After centrifugation, 6 fractions were collected from top to bottom of the gradient. For protein detection, samples were boiled in SDS loading buffer prior to SDS-PAGE and western blotting.

### RNA and protein analysis

Total yeast RNA isolation by the hot phenol method, Northern blot analysis, total protein extractions and Western blot analysis, and anti-1a, anti-2a^pol^, anti-Dpm1, anti-FLAG and anti-Pgk1 antibodies were as described [4, 15, 81, 82]. Mouse anti-PDI was acquired from Abcam, rabbit and mouse anti-HA antibodies were purchased from Santa Cruz and Roche, respectively. Mouse anti-GFP was purchased from Covance. Northern blots were imaged on a Typhoon 9200 (Amersham Biosciences, Piscataway, NJ). Band intensities were analyzed by using ImageQuant software (Molecular Dynamics, Piscataway, NJ).

### Coimmunoprecipitation

Yeast cells were lysed in RIPA buffer (1% NP-40, 0.1% SDS, 50 mM Tris pH 8.0, 150 mM NaCl, 0.5% Sodium deoxycholate, 5 mM EDTA, 10 mM NaF, 10 mM NaPPi, 2 mM phenylmethylsulfonyl, 5mM benzamidine, and 10 ug/ml each of chymostatin, pepstatin A, leupeptin, bestatin) using glass beads and a bead beater and the supernatant was collected after centrifugation. For immunoprecipitation, yeast lysates were mixed with Protein A Sepharose beads (GE Healthcare. Piscataway, NJ) and anti-1a, anti-HA, or anti-GFP antibodies overnight at 4°C. Beads were pelleted and washed with RIPA buffer before boiling in 1x SDS gel loading buffer and running samples in 4–15% SDS-PAGE gels.

### Immunofluorescence and confocal microscopy

Confocal microscopy was as described [80]. Briefly, to detect the subcellular localization of BMV proteins in ESCRT deletion strains, yeast were transformed with plasmids expressing BMV 1a and 2a^pol^ proteins. To detect the localization of the ESCRT proteins, HA-tagged ESCRTs were transformed with empty plasmids or plasmids encoding BMV 1a. Cells were fixed with 4% formaldehyde, spheroplasted with lyticase, and permeabilized with 0.1% Triton X-100. Spheroplasts were then stained either by using rabbit anti-1a serum, mouse anti-PDI, mouse anti-HA antibodies or combinations thereof followed by anti-rabbit or anti-mouse secondary antibodies conjugated to Alexa-488, or Alexa-568. For nuclear staining, a 10-minute incubation with 300 nM DAPI (Invitrogen) was added after secondary antibody incubation. Fluorescent images were acquired with a Nikon A1R Bio-Rad inverted confocal microscope system. To avoid spectral bleed-through in the multi-color imaging, images were acquired by sequentially scanning with the individual lasers and detecting fluorescence in each channel to coincide with laser illumination.

### Electron microscopy

Samples were prepared for electron microscopy as described [22]. In brief, yeast cells were fixed for 1 hr with 2% glutaraldehyde and 4% paraformaldehyde, washed, and post-fixed for 1 hr with 1% OsO4 and 1% uranyl acetate. Cells then were dehydrated via a series of step-wise increasing ethanol concentrations ranging from 50% to 100%, and infiltrated and embedded with Spurr’s resin. Samples were sectioned and placed on nickel grids, washed, and incubated for 15 min in 2% glutaraldehyde, post-stained with 8% uranyl acetate and Reynold's lead citrate, and viewed with a Philips CM120 microscope. For each deletion strain, the diameter of >50 spherules were measured with the imaging program ITEM Analysis (Soft Imaging Systems, Lakewood, Colo.).

## Supporting Information

S1 Fig BMV RNA replication is not affected in the absence of *HSE1* or *VPS36*. Total RNA extracts were obtained from wt, *hse1Δ*, or *vps36Δ* deletion strains expressing 1a, 2a^pol^, and RNA3 and accumulation of negative-strand RNA3 and positive-strand subgenomic RNA4 was measured by Northern blotting. Equal loading was verified by probing for 18S ribosomal RNA. Values represent the mean of four independent experiments, with each condition tested in triplicate in each experiment. A representative blot is shown. All samples were compared to the wt control using a two tailed student t-test. ns: not significant.
(EPS)
