# Correction: Host ESCRT Proteins Are Required for Bromovirus RNA Replication Compartment Assembly and Function

**DOI:** 10.1371/journal.ppat.1004845

**Published:** 2015-04-22

**Authors:** Arturo Diaz, Jiantao Zhang, Abigail Ollwerther, Xiaofeng Wang, Paul Ahlquist

The following information is missing from the Funding section: Work in XW's lab was also supported by the Virginia Agricultural Experiment Station and the Hatch Program of NIFA, USDA.
